# Is Proflavine Exposure Associated with Disease Progression in Women with Cervical Dysplasia? A Brief Report

**DOI:** 10.1111/php.12976

**Published:** 2018-07-31

**Authors:** Naitielle Pantano, Brady Hunt, Richard A. Schwarz, Sonia Parra, Katelin Cherry, Júlio César Possati‐Resende, Adhemar Longatto‐Filho, José Humberto Tavares Guerreiro Fregnani, Philip E. Castle, Kathleen Schmeler, Rebecca Richards‐Kortum

**Affiliations:** ^1^ Institute of Education and Research Barretos Cancer Hospital Pio XII Foundation Barretos São Paulo Brazil; ^2^ Molecular Oncology Center Department of Cancer Prevention Barretos Cancer Hospital Pio XII Foundation Barretos São Paulo Brazil; ^3^ Department of Bioengineering Rice University Houston TX; ^4^ Faculty of Medicine Laboratory of Medical Investigation (LIM) 14 FMUSP São Paulo University São Paulo Brazil; ^5^ School of Health Sciences Life and Health Sciences Research Institute ICVS Uminho University Braga Portugal; ^6^ ICVS/3B's ‐ PT Government Associate Laboratory Braga/Guimarães Portugal; ^7^ Global Coalition against Cervical Cancer New York NY; ^8^ Department of Epidemiology and Population Health Albert Einstein College of Medicine Bronx NY; ^9^ Department of Gynecologic Oncology and Reproductive Medicine The University of Texas MD Anderson Cancer Center Houston TX

## Abstract

Proflavine is an acridine dye used with high‐resolution microendoscopy for *in vivo* diagnostic evaluation of cervical epithelial cells. However, there are concerns that even short‐term exposure of cervical tissue to dilute proflavine may increase cervical cancer risk. We performed a retrospective analysis of women referred for colposcopy to Barretos Cancer Hospital comparing the risk of cervical disease progression in those whose cervical tissue was (*n* = 232) or was not exposed (*n* = 160) to proflavine. Patients in both groups underwent treatment and follow‐up based on histopathologic results and per the local standards of care. Progression of disease was evaluated by comparing histopathology from the initial visit to the worst subsequent histopathology result from all follow‐up visits. Mean duration of follow‐up was 18.7 and 20.1 months for the proflavine‐exposed and controls groups, respectively. There were no significant differences in disease progression from normal/CIN1 to CIN2/3 or from any initial diagnosis to invasive cancer between the proflavine exposed and control groups overall. Risks of cervical dysplasia progression observed in this study are in agreement with those of the natural history of cervical cancer. Our results suggest that cervical exposure to dilute proflavine does not increase the risk of cervical precancer and cancer.

## Introduction

Proflavine is a fluorescent dye that has long been recognized and used as a topical antibacterial agent. Its history of use as an antiseptic in wound care dates back to the early 1900s, and its action as a nucleic acid intercalator has been established as the basis of its antibacterial properties 1, 2. Today proflavine continues to be used as a commercially available antiseptic in many parts of the world 3, 4. Proflavine is one of the components of triple dye, which is routinely used for umbilical cord care in newborn infants in the United States 5, 6. Toxicity of triple dye is rare 6.

With the development of *in vivo* fiber‐optic microscopy techniques over the past two decades, proflavine and acriflavine (another closely related acridine compound) have been frequently used as topical contrast agents for optical imaging due to their ability to fluorescently label cell nuclei with high contrast. Acriflavine has been reported as a contrast agent for *in vivo* confocal laser endomicroscopy in the colon 7, 8, stomach 9, duodenum 10, upper gastrointestinal tract 11 and central airway 12. In these studies, acriflavine was applied topically to the tissue prior to imaging, usually in combination with intravenous fluorescein. Our group has reported the use of topically applied proflavine as a contrast agent for *in vivo* microscopy in the oral cavity 13, esophagus 14 and cervix 15. The use of proflavine or acriflavine in combination with *in vivo* microscopy enables real‐time assessment of the morphology and distribution of cell nuclei, aiding in the accurate identification of precancerous lesions 16.

Despite their long history of safe clinical use, there remains disagreement on the cancer risk of using proflavine and acriflavine as contrast agents for *in vivo* imaging. In 1977, the National Cancer Institute published a bioassay of proflavine for possible carcinogenicity, based on administration of proflavine in the diet to groups of rats and mice over a 2‐year period 17. The bioassay was inconclusive due to an unusually high incidence of carcinomas in control animals. The International Agency for Research on Cancer (IARC) published evaluations of the carcinogenic risk of acriflavine in 1977 18 and of proflavine in 1980 19. These evaluations concluded that while acriflavine and proflavine display mutagenicity *in vitro*, their carcinogenic risk to humans could not be classified due to inadequate data in experimental animals and a lack of data in humans. Some view the mutagenicity of these compounds *in vitro* as sufficient reason for concern. For example, a recent publication recommends the use of methylene blue over acriflavine as an imaging contrast agent, due to concerns about the *in vitro* mutagenicity of acriflavine 20. However, other recent publications suggest that acriflavine and proflavine exhibit beneficial anticancer and antiviral effects. Acriflavine has been shown to reduce tumor growth in mice by inhibiting HIF‐1 dimerization 21. An acriflavine and proflavine mixture has been shown to elicit an antiviral immune response that significantly reduced rhinovirus infection in mammalian cells 22. At this time, there is no evidence demonstrating the carcinogenicity of proflavine or acriflavine in human subjects, and as such, both compounds remain categorized by the IARC as “not classifiable as to its carcinogenicity to humans” 23, 24.

Yet concerns remain. Therefore, we conducted a retrospective study to evaluate whether proflavine exposure is associated with progression of cervical neoplasia in women with abnormal cervical cytology by comparing the rate of cervical intraepithelial neoplasia progression between two groups, one exposed to proflavine and the other not exposed.

## Materials and methods

### *Study participants*

This is a historical cohort study. The proflavine group was composed of women who had previously participated in a clinical trial (ID# NCT02335372) at Barretos Cancer Hospital (Brazil) between June 2013 and January 2015. Participants in the trial were recruited from women presenting for colposcopy due to abnormal cytology or previous history of cervical dysplasia at the Prevention Department of Barretos Cancer Hospital. This trial evaluated a new optical imaging device, the high‐resolution microendoscope (HRME), a portable, battery‐powered fluorescence microscope with a flexible fiber‐optic probe 25, 26, 27. The details of this experimental device have previously been described 15. As part of the study protocol, 5% acetic acid was applied to the surface of the uterine cervix and standard of care colposcopy was performed. This was followed by application of 5% Lugol's iodine solution. Proflavine solution at a concentration of 0.01% was then applied to the cervix followed by evaluation with the HRME probe. The typical volume dispensed by the spray bottle was measured to be 3 mL with a range of 1–7 mL. The control group was identified retrospectively and was balanced for severity of baseline cytology diagnosis prior to colposcopy. The control group comprised of women who underwent colposcopy and cervical biopsies in the Prevention Department of Barretos Cancer hospital between May 2013 and May 2016. Patients in the control group did not undergo evaluation with HRME and thus were not exposed to proflavine; however, they were subjected to equivalent diagnostic, treatment and follow‐up procedures. This retrospective study was approved by the Barretos Cancer Hospital Ethics Research Committee, the Brazilian National Ethics Research Commission (CAAE: 83227718.8.0000.5437).

### Follow‐up and treatment

Follow‐up data including all histopathology results were collected for patients in both groups from the time of the initial colposcopy until the last appointment available from the medical record. In both groups, the treatment and follow‐up were performed according to the local standard of care based on histopathology results. Those women diagnosed with cervical intraepithelial neoplasia grades 2 (CIN 2) or 3 (CIN 3) underwent treatment with loop electrosurgical excision procedure (LEEP). Those diagnosed with invasive cancer were referred to the Gynecologic Oncology department for care.

### Statistical analysis

Data for all patients in the proflavine and control groups were collected from electronic medical records at Barretos Cancer Hospital and compiled into an SPSS database file. The database consisted of a total 499 patient records with 299 records in the proflavine group and 200 in the control group. The following data fields were aggregated for each participant: age, study group, initial cytology result, date of initial colposcopy, initial histopathology result, date of last clinical visit and up to six subsequent histopathology results. For all histopathology results, the date of diagnosis and tissue specimen type (cervical biopsy, endocervical curettage (ECC), LEEP, or hysterectomy) were documented. The total follow‐up time period was calculated as the time between the first and last colposcopy evaluations on record.

Cervical cytology results were grouped into two categories: normal/low grade and high grade. Normal/low grade included the following: negative for intraepithelial lesions or malignancy (NILM), atypical squamous cells of undetermined significance (ASC‐US) and low‐grade squamous intraepithelial lesion (LSIL). High‐grade cytology results included: high‐grade squamous intraepithelial lesion (HSIL), atypical squamous cells cannot exclude high‐grade squamous intraepithelial lesion (ASC‐H), atypical glandular cells (AGC), squamous cell carcinoma and adenocarcinoma. In order to assess cervical dysplasia progression, histopathology results were grouped into five clinically relevant categories by increasing severity of diagnosis: negative for intraepithelial lesion (NIL), cervical intraepithelial neoplasia grade 1 (CIN 1), CIN 2 or CIN 3 (CIN 2/3), adenocarcinoma *in‐situ* (AIS) and invasive cancer. NIL category included diagnoses of normal, inflammation, hyperplasia and metaplasia. CIN 2/3 category included diagnoses of CIN 2, CIN 3 and CIN 2/3. Invasive cancer category included diagnoses of invasive squamous cell carcinoma and adenocarcinoma. In cases where follow‐up colposcopy was negative and no biopsies were obtained, the visit was categorized as normal colposcopy/no biopsy performed.

A total of 107 patients were excluded from the analysis for the following reasons: (1) total follow‐up duration was less than 6 months (proflavine group, *n* = 49; control group, *n* = 29), and (2) patient had an initial histological diagnosis of invasive carcinoma (proflavine group, *n* = 18; control group, *n* = 11). After applying these exclusion criteria, 392 patients were included in the retrospective analysis (proflavine group, *n* = 232; control group, *n* = 160). Using these data, the study population was characterized using descriptive statistics. Categorical variables were compared using Fisher's exact test, and continuous numerical variables were compared using Student's t test. Survival analysis was performed on two clinical endpoints: (1) progression from a baseline histopathologic diagnosis of <CIN2 to a subsequent histopathologic diagnosis of CIN2+, (2) treatment via LEEP. A spreadsheet containing all patients was compiled with the following information: cohort (proflavine or control), time from baseline diagnosis to the event of interest (days), and the status (1 = event occurred, 0 = event not occurred) at the indicated time interval. A survival function was fit using the “survival” package of the R programming language 28. The functions for both proflavine and control cohorts were plotted over time and the resulting fits compared using the G‐rho family of tests 29. Statistical significance level was set at 5%. *Post hoc* statistical power was calculated using PASS (NCSS LLC, Kaysville, UT, USA).

## Results

### Baseline patient characteristics

There were no statistically significant differences between groups according to their initial diagnosis by cytology and/or histopathology from cervical biopsies (Table 1). The mean age at diagnosis was slightly higher in the control arm (mean = 37.8 years; SD = 13.0 years) in comparison with the proflavine arm (mean = 35.8 years; SD = 11.7 years) but not statistically significant (*P* = 0.10). The mean time of follow‐up was 18.7 months (SD = 6.0 months) and 20.1 months (SD = 5.6 months) for the proflavine and control groups, respectively.

**Table 1:** Baseline diagnosis according to the study group

	Study Group		*P*‐value*
	Proflavine Exposed (*n* = 232, %)	Control (*n* = 160, %)	
Cytology			
Normal/low‐grade	54 (23.3)	36 (22.5)	0.90
High‐grade	178 (76.7)	124 (77.5)	
Histopathology			
<CIN2	125 (53.9)	77 (48.1)	0.30
CIN2+	107 (46.1)	83 (51.9)	

Normal/low grade = NILM, ASC‐US, LSIL; High grade = ASC‐H, AGC, HSIL, Carcinoma, Adenocarcinoma; < CIN2 = Cervicitis, Hyperplasia, Metaplasia, CIN1, CIN2+ = CIN 2, CIN 3, Adenocarcinoma *In‐situ*, Carcinoma, Adenocarcinoma. *P‐values calculated using Fisher's exact test.

### Cervical dysplasia progression

In order to evaluate cervical dysplasia progression, we compared initial and subsequent worst histopathologic diagnoses for the patients exposed to proflavine (*n* = 232) and those not exposed (*n* = 160). Because removal of proflavine‐exposed tissue via LEEP may mitigate the effect of proflavine exposure, we stratified our analysis within each cohort by those who underwent LEEP and those who did not. A LEEP was performed in 124 of the 232 (53%) patients exposed to proflavine and in 96 of the 160 (60%) control patients (*P* = 0.21).

Table 2 shows the initial and subsequent worst diagnoses for patients who did not undergo LEEP in the proflavine and control groups, respectively. There were a total of nine patients that had benign histopathology who had a subsequent diagnosis of CIN 1 (proflavine 5%; control 6%; *P* = 0.73). Additionally, there was one patient in the control group that progressed from an initial diagnosis of CIN 1 to CIN 2/3 (proflavine 0%; control 2%). The majority of non‐LEEP patients had no abnormal cervical lesions observed during follow‐up colposcopy, and thus, no additional cervical biopsies were required (proflavine 52%; control 58%; *P* = 0.53).

**Table 2:** Initial and worst subsequent pathologic diagnoses for patients who did not undergo LEEP

	Exposed to Proflavine and did not Undergo LEEP (*n* = 108/232, 47%)							Not Exposed to Proflavine and did not Undergo LEEP (*n* = 64/160, 40%)						
	Worst Subsequent Pathologic Diagnosis							Worst Subsequent Pathologic Diagnosis						
	Normal Colposcopy/No Biopsy(%)	NIL (%)	CIN 1 (%)	CIN 2/3 (%)	AIS (%)	Invasive Cancer (%)	Total (%)	Normal Colposcopy/No Biopsy (%)	NIL (%)	CIN 1 (%)	CIN 2/3 (%)	AIS (%)	Invasive Cancer (%)	Total (%)
Initial Pathologic Diagnosis														
NIL	10 (9)	2 (2)	5 (5)	0 (0)	0 (0)	0 (0)	17 (16)	17 (27)	7 (11)	4 (6)	0 (0)	0 (0)	0 (0)	28 (44)
CIN 1	45 (42)	12 (11)	28 (26)	0 (0)	0 (0)	0 (0)	85 (79)	19 (30)	2 (3)	7 (11)	1 (2)	0 (0)	0 (0)	29 (45)
CIN 2/3	1 (1)	0 (0)	2 (2)	3 (3)	0 (0)	0 (0)	6 (6)	1 (2)	1 (2)	1 (2)	4 (6)	0 (0)	0 (0)	7 (11)
AIS	0 (0)	0 (0)	0 (0)	0 (0)	0 (0)	0 (0)	0 (0)	0 (0)	0 (0)	0 (0)	0 (0)	0 (0)	0 (0)	0 (0)
Total	56 (52)	14 (13)	35 (32)	3 (3)	0 (0)	0 (0)	108 (100)	37 (58)	10 (16)	12 (19)	5 (8)	0 (0)	0 (0)	64 (100)

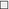
, Cases where subsequent pathology was equal to initial; 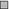, Cases where subsequent pathology was worse than initial. Normal Colposcopy/No Biopsy = Follow‐up colposcopy examinations were normal and did not require additional biopsies; NIL = Negative for Intraepithelial Lesion (normal, inflammation, hyperplasia and metaplasia); CIN 1 = Cervical Intraepithelial Neoplasia Grade 1; CIN 2/3 = Cervical Intraepithelial Neoplasia Grades 2, 3 and 2/3; AIS = Adenocarcinoma *In‐situ*; Invasive Cancer = Invasive adenocarcinoma.

Table 3 provides a similar summary of initial and final histopathology for patients in both study groups that did undergo LEEP during the follow‐up period. The most common initial biopsy diagnosis in both patient populations was CIN 2/3, which was subsequently equally confirmed by the diagnosis on the LEEP tissue (proflavine 65%; control 66%; *P* = 1.0). There were 28 patients with benign initial histopathology on biopsy who had a worst subsequent diagnosis of CIN 2/3 on the LEEP tissue (proflavine 12%; control 13%). In addition, three patients in the control group had benign initial histopathology with a worst subsequent diagnosis of CIN 1 (proflavine 0%; control 3%). One invasive cervical cancer was diagnosed both in the proflavine exposed and control groups.

**Table 3:** Initial and worst subsequent pathologic diagnoses for patients who underwent LEEP

	Exposed to Proflavine and Underwent LEEP (%)(*n* = 124/232, 53%)							Not Exposed to Proflavine and Underwent LEEP (%)(*n* = 96/160, 60%)						
	Worst Subsequent Pathologic Diagnosis							Worst Subsequent Pathologic Diagnosis						
	Normal Colposcopy/No Biopsy(%)	NIL (%)	CIN 1 (%)	CIN 2/3 (%)	AIS (%)	Invasive Cancer (%)	Total (%)	Normal Colposcopy/No Biopsy (%)	NIL (%)	CIN 1 (%)	CIN 2/3 (%)	AIS (%)	Invasive Cancer (%)	Total (%)
Initial Pathologic Diagnosis														
NIL	0 (0)	1 (1)	0 (0)	6 (5)	0 (0)	0 (0)	7 (6)	0 (0)	0 (0)	3 (3)	4 (4)	0 (0)	1 (1)	8 (8)
CIN 1	0 (0)	0 (0)	7 (6)	9 (7)	0 (0)	0 (0)	16 (13)	0 (0)	0 (0)	3 (3)	9 (9)	0 (0)	0 (0)	12 (13)
CIN 2/3	0 (0)	8 (6)	11 (9)	81 (65)	0 (0)	0 (0)	100 (81)	0 (0)	5 (5)	8 (8)	63 (66)	0 (0)	0 (0)	76 (79)
AIS	0 (0)	0 (0)	0 (0)	0 (0)	0 (0)	1 (1)	1 (1)	0 (0)	0 (0)	0 (0)	0 (0)	0 (0)	0 (0)	0 (0)
Total	0 (0)	9 (7)	18 (15)	96 (77)	0 (0)	1 (1)	124 (100)	0 (0)	5 (5)	14 (15)	76 (79)	0 (0)	1 (1)	96 (100)

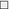
, Cases where subsequent pathology was equal to initial; 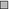, Cases where subsequent pathology was worse than initial; Normal Colposcopy/No Biopsy = Follow‐up colposcopy examinations were normal and did not require additional biopsies; NIL = Negative for Intraepithelial Lesion (normal, inflammation, hyperplasia and metaplasia); CIN 1 = Cervical Intraepithelial Neoplasia Grade 1; CIN 2/3 = Cervical Intraepithelial Neoplasia Grades 2, 3 and 2/3; AIS = Adenocarcinoma *In‐situ*; Invasive Cancer = Invasive adenocarincoma.

The risks of cervical dysplasia progression for all groups are summarized in Table 4. Progression from normal/CIN1 to CIN 2/3 was noted in 15/232 (6.5%) proflavine‐exposed patients and 14/160 (8.8%) of controls (*P* = 0.44). Progression from any initial diagnosis to invasive cancer was noted in one of 232 patients exposed to proflavine (0.4%) and one of 160 controls (0.6%) (*P* = 1.0). Based on the total number of cases where subsequent pathology was worse than initial, there were no statistically significant differences in progression of cervical dysplasia between proflavine‐exposed and nonexposed patients (Table 4) (all patients: *P* = 0.19; non‐LEEP patients: *P* = 0.50; LEEP patients: *P* = 0.35).

**Table 4:** Summary of cases where subsequent pathologic diagnosis was worse than baseline diagnosis

		All Patients			Did Not Undergo LEEP			Underwent LEEP		
Initial Diagnosis	Worst Subsequent Diagnosis	Proflavine Exposure (*n* = 232) (%)	No Exposure (*n* = 160) (%)	*P*‐value^a^	Proflavine Exposure (*n* = 108) (%)	No Exposure (*n* = 64) (%)	*P*‐value	Proflavine Exposure (*n* = 124) (%)	No Exposure (*n* = 96) (%)	*P*‐value
NIL	CIN 1	5 (2.2)	7 (4.4)	0.19	5 (4.6)	4 (6.3)	0.50	0 (0)	3 (3.1)	0.35
NIL	CIN 2/3	6 (2.6)	4 (2.5)		0 (0)	0 (0)		6 (4.8)	4 (4.2)	
CIN 1	CIN 2/3	9 (3.9)	10 (6.3)		0 (0)	1 (1.6)		9 (7.3)	9 (9.4)	
NIL	Invasive Cancer	0 (0)	1 (0.6)		0 (0)	0 (0)		0 (0)	1 (1.0)	
AIS	Invasive Cancer	1 (0.4)	0 (0)		0 (0)	0 (0)		1 (0.8)	0 (0)	
Total		21 (9.1)	22 (13.8)		5 (4.6)	5 (7.8)		16 (12.9)	17 (17.7)	

a *P*‐values calculated using Fisher's exact test based on total cases where subsequent diagnosis was worse than initial.

**Figure 1:**
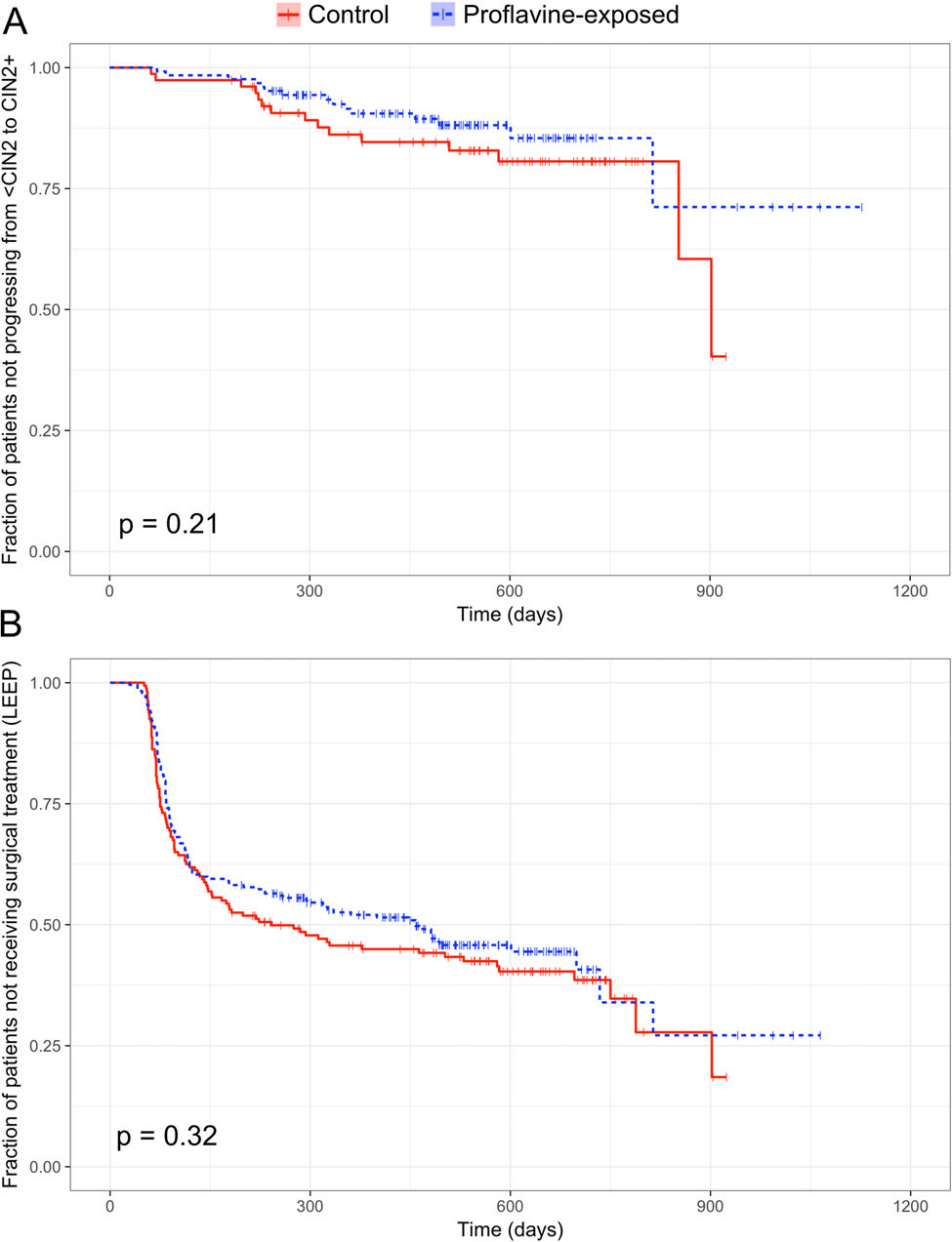
Kaplan–Meier estimate of (a) progression from <CIN2 to CIN2 + and (b) surgical treatment (LEEP). Vertical tick marks represent censoring at the indicated time point. Statistical comparisons were performed using a log‐rank test.

Figure 1 shows a Kaplan–Meier estimate of two clinical endpoints: progression from a baseline histopathologic diagnosis of <CIN2 to a subsequent histopathologic diagnosis of CIN2+ (proflavine group, *n* = 125; control group, *n* = 77) and treatment via LEEP (proflavine group, *n* = 232; control group, *n* = 160). No statistically significant difference was found for progression of histological diagnosis from <CIN2 to CIN2+ between proflavine‐exposed and control groups (log‐rank test, *p* = 0.21). Additionally, no statistically significant difference was found for treatment by LEEP between proflavine‐exposed and control groups (log‐rank test, *P* = 0.32).

## Discussion

In this study, we assessed whether proflavine might increase the risk of cervical neoplasia progression when used as a fluorescent contrast agent for *in vivo* microscopy during colposcopic examination. Our results showed no significant increases in cervical dysplasia progression in women exposed to proflavine when compared to a control group of nonexposed women. This is important for routine gynecological examinations because proflavine can be used as a diagnostic tool in conjunction with colposcopy, facilitating the recognition of cervical lesions through *in vivo* microscopy as previously reported by Grant *et al*. 15. The primary contribution of this study is to provide initial data regarding the use of proflavine in human subjects with cervical dysplasia. We are not aware of any prior studies assessing proflavine and cervical dysplasia progression; however, these results may also be put into context of other studies investigating the natural history of cervical dysplasia.

In a meta‐analysis of 17 studies involving 4504 participants, Östӧr 30 found that 10% of women with CIN 1 will progress to CIN 3 and only 1% will progress to invasive cancer. Follow‐up durations in this meta‐analysis varied widely from as little as 3 weeks up to 25 years 30. A more recent analysis of 524 patients from the placebo arm of the quadrivalent HPV vaccine trials found that 12% of patients with initial diagnosis of CIN 1 developed CIN 2/3 during follow‐up 31. The findings in this study are in agreement with previously established risks.

The total follow‐up duration achieved by this analysis is one limitation of the study. Even with this limitation, the structure of this study provides a meaningful comparison for cervical dysplasia progression in proflavine‐exposed and nonexposed subjects. The *post hoc* statistical power was ≥80% (*α* = 0.05) to detect an absolute increase in risk of upstaging from <CIN2 to CIN2+ of 18% (or more). However, longer‐term follow‐up studies with larger sample sizes will be necessary to provide reassurance for the topical use of proflavine on the cervical epithelium.

In conclusion, the risks of cervical dysplasia progression observed in this study are in agreement with those of the natural history of cervical cancer. Our findings suggest that cervical proflavine exposure is not associated with acute disease progression in women with cervical dysplasia.
